# Functional dynamics of a single tryptophan residue in a BLUF protein revealed by fluorescence spectroscopy

**DOI:** 10.1038/s41598-020-59073-5

**Published:** 2020-02-06

**Authors:** Kristof Karadi, Sofia M. Kapetanaki, Katalin Raics, Ildiko Pecsi, Robert Kapronczai, Zsuzsanna Fekete, James N. Iuliano, Jinnette Tolentino Collado, Agnieszka A. Gil, Jozsef Orban, Miklos Nyitrai, Greg M. Greetham, Marten H. Vos, Peter J. Tonge, Stephen R. Meech, Andras Lukacs

**Affiliations:** 1 Department of Biophysics, Medical School, University of Pécs, 7624 Pécs, Hungary; 2 Szentagothai Research Center, University of Pécs, 7624 Pécs, Hungary; 3 Department of Chemistry, Stony Brook University, Stony Brook, NY 11794-3400 USA; 4 Central Laser Facility, Harwell Science and Innovation Campus, Didcot, Oxfordshire UK; 5 LOB, CNRS, INSERM, Ecole Polytechnique, Institut Polytechnique de Paris, 91128 Palaiseau, Cedex France; 6 School of Chemistry, University of East Anglia, Norwich, NR4 7TJ UK

**Keywords:** Biochemistry, Biophysics

## Abstract

Blue Light Using Flavin (BLUF) domains are increasingly being adopted for use in optogenetic constructs. Despite this, much remains to be resolved on the mechanism of their activation. The advent of unnatural amino acid mutagenesis opens up a new toolbox for the study of protein structural dynamics. The tryptophan analogue, 7-aza-Trp (7AW) was incorporated in the BLUF domain of the Activation of Photopigment and pucA (AppA) photoreceptor in order to investigate the functional dynamics of the crucial W104 residue during photoactivation of the protein. The 7-aza modification to Trp makes selective excitation possible using 310 nm excitation and 380 nm emission, separating the signals of interest from other Trp and Tyr residues. We used Förster energy transfer (FRET) between 7AW and the flavin to estimate the distance between Trp and flavin in both the light- and dark-adapted states in solution. Nanosecond fluorescence anisotropy decay and picosecond fluorescence lifetime measurements for the flavin revealed a rather dynamic picture for the tryptophan residue. In the dark-adapted state, the major population of W104 is pointing away from the flavin and can move freely, in contrast to previous results reported in the literature. Upon blue-light excitation, the dominant tryptophan population is reorganized, moves closer to the flavin occupying a rigidly bound state participating in the hydrogen-bond network around the flavin molecule.

## Introduction

**Figure 1:**
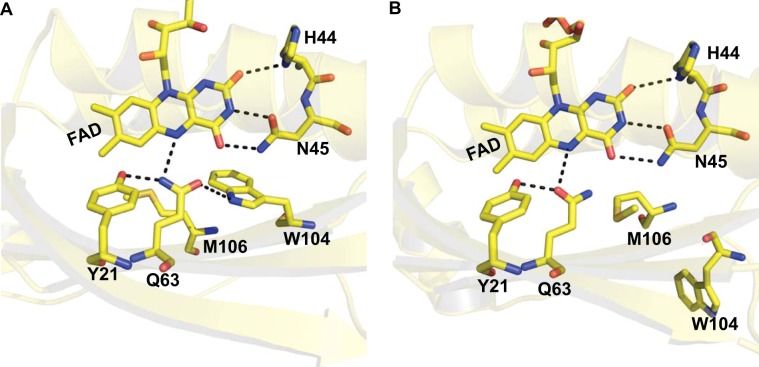
Ribbon diagrams of the BLUF domain crystal structures from WT (A, pdb:1yrx) and C20S (B, pdb:2iyg) AppA_BLUF_ domains showing W104 in the vicinity of the flavin in the Trp_*in*_ conformation (**A**) and away from the flavin in the Trp_*out*_ conformation (**B**). Important residues involved in an H-bond network around the flavin (Y21, H44, N45, Q63) are shown. Dashed lines represent H-bonds between the flavin and the residues.

Flavins are found in more than 370 enzymes^1^ but only a few of them are photoactive^2,3^. Three major families of photoreceptors which utilize flavin as a cofactor and whose functions are triggered by absorption of light are the photolyase/cryptochromes, the light oxygen voltage (LOV) domains and the blue light sensors using flavin (BLUF) proteins. Their photochemistry, though is rather diverse. In photolyases and cryptochromes, FAD (flavin adenine dinucleotide) is reduced via electron transfer through a tryptophan triad^4–6^. Photolyases use light to repair UV-damaged DNA^7^ whereas the proposed functions of cryptochromes range from setting the circadian clock in insects to sensing the weak magnetic field of Earth in migrating birds^6^. In the LOV domains, the flavin cofactor is excited to a triplet state upon blue light absorption, followed by formation of a signalling state, characterized by a covalent bond between the flavin and a nearby cysteine residue, leading to the enhancement of the phototropin kinase activity^3^. In BLUF domains, blue light excitation results in a signalling state (light-adapted state) that is characterized by a reorganization of the hydrogen bond network around FAD and the Tyr-Gln-Trp (Met) tetrad (Fig. 1). This is revealed by a characteristic 10–15 nm red-shift of the first π → π* transition and a 20 cm^−1^ downshift of the flavin C4=O stretching vibration compared to the dark-adapted state^8,9^. In AppA_BLUF_,  site directed mutagenesis has shown that Y21 and Q63 play a crucial role during photoactivation as the red shift upon illumination disappears if one of these residues is replaced^8–12^. A strong and specific chemical coupling between the flavin and the protein by means of glutamine tautomerization has been proposed to provide a basis for light sensing^13^.

W104 is also a key player in the photocycle in communicating the electronic excitation of the flavin ring to the protein backbone^14^. Recent time-resolved IR experiments^15^ have shown that W104A AppA_BLUF_ undergoes reduced structural changes on the µs timescale along with an 80-fold increase in the rate of dark-state recovery, compared to that of the wild-type^16^. A 1.5-fold increase of the quantum yield of signalling state formation with a slight increase of the ground state recovery have been observed for the W104F AppA_BLUF_ mutant^17^. Mutation of the W104 residue (W104A) does suppress the red-shift in the full-length protein^18^ and renders the protein insensitive to blue light *in vivo*^16^ underlying the importance of this residue for the downstream signalling events.

The exact conformation of W104 during the photoactivation process in AppA and other BLUF domains has been a controversial topic in the field. The first crystal structure of AppA^19^ (pdb:1yrx) showed that W104 in the dark-adapted state is located close to flavin in the so called Trp_*in*_ conformation (Fig. 1A), whereas subsequent crystal structures from Schlichting and coworkers^20^ (pdb: 2iyg, 2iyi) presented a different picture where the tryptophan is pointing away from the flavin both in the dark- and light-adapted state (Trp_*out*_ conformation) (Fig. 1B). Crystal structures of other BLUF domains like PixD^21,22^, BlrB^23^ and OaPAC^24^ have also contributed to the confusion as tryptophan is located in a solvent exposed position in a Trp_*out*_ conformation. Interestingly, Slr1694 exhibits both conformations with one of the 10 crystallographic subunits adopting the Trp_*in*_ conformation and the others the Trp_*out*_ conformation^22^.

Computational calculations and spectroscopic studies have tried to address the question of the tryptophan flip in AppA, leading to opposite conclusions. Two independent studies by Udvarhelyi et *al*.^25^ and Collette et *al*.^26^ concluded that the Trp_*out*_ represents the dark-adapted state of the protein whereas a recent study by Goyal and Hammes-Schiffer^27^ revealed that both the *in* and *out* conformations are allowed at equilibrium but the *in* conformation is more stable. The solution NMR structure (pdb: 2bun) has also the majority of the snapshots in the Trp_*in*_ conformation^28^. UV resonance Raman studies have supported the Trp_*in*_ conformation in the dark state^29^. Tryptophan fluorescence spectroscopic studies have suggested that Trp does not become fully solvent exposed as suggested by some crystal structures but were conflicting regarding the conformation of the Trp^18,30^.

As there is still a controversy about the conformation and the role of W104 during photoactivation of AppA, we revisited the question using a different approach. Specifically, we exploit the spectroscopic properties of the 7-aza Trp (7AW) unnatural amino acid as an intrinsic fluorescent probe, able to separate the signals of interest from other Trp and Tyr residues. The absorption and fluorescence maxima of 7AW are red-shifted compared to canonical Trp allowing the exclusive excitation of the Trp analogue. We incorporated 7AW at position 104 and applied a wide range of fluorescence techniques to probe the functional dynamics of Trp104 in AppA_BLUF_. The results of our study provide strong evidence that W104 adopts a Trp_*out*_ conformation in the dark-adapted state and a Trp_*in*_ conformation in the light-adapted state.

## Results

### FRET measurements

To provide quantitative information on the position of Trp104 during the photoactivation process in AppA, we used fluorescence resonance energy transfer (FRET) measurements.

Fluorescence resonance energy transfer, is a long-range non-radiative energy transfer process that takes place between two fluorophores, one in the excited state and one in the ground state^31,32^. The FRET efficiency-which is defined as the fraction of energy absorbed by the donor that is subsequently transferred to the acceptor depends on the distance between the donor and the acceptor^33,34^ and can be calculated by Eq. (1)

1 $$E=\frac{1}{1+{(\frac{R}{R0})}^{6}}$$

where *E* is the FRET efficiency, *R* is the distance between the donor and the acceptor fluorophores and *R*_0_ is the distance between the two fluorophores in the case of 50% transfer efficiency. This strong distance dependence of FRET efficiency makes possible the measurement of molecular distances in the range of 10–100 Å and the term “spectroscopic ruler” has been coined for the method^35^.

**Figure 2:**
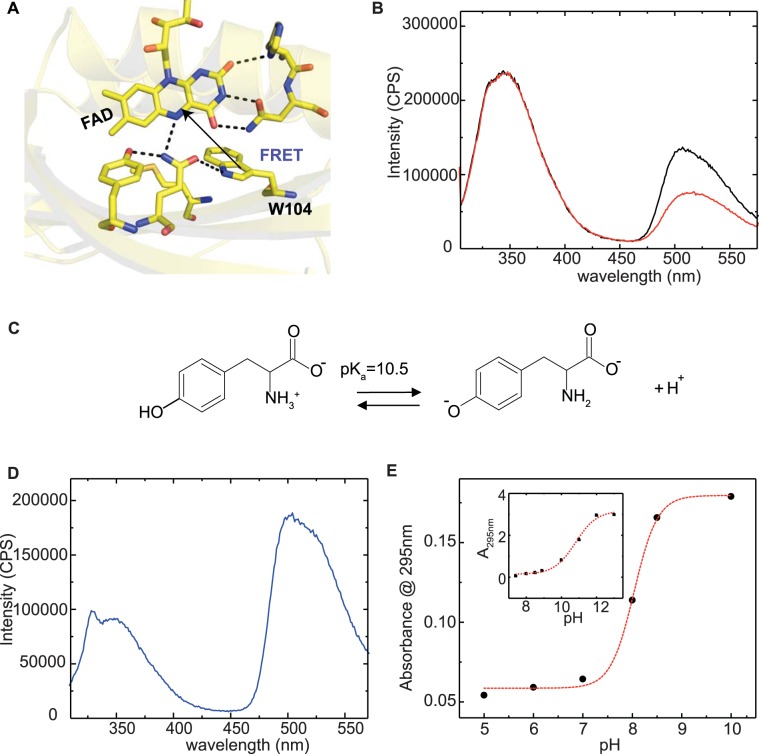
(**A**) Ribbon diagram of the crystal structure of C20S AppA_BLUF_ (pdb: 1yrx), showing the FRET pair (FAD, W104). (**B**) Emission spectra of W64F AppA_BLUF_ in the dark- (black) and light- (red) adapted states (λ_exc_ = 295 nm). (**C**) Chemical structures showing ionization of tyrosine to tyrosinate. (**D**) Emission spectrum of W64F/W104A AppA_BLUF_ (λ_exc_ = 295 nm). The narrow peak ~330 nm is the contribution of Raman scattering of water (**E**) pH dependent formation of tyrosinates monitored at 295 nm for the W64F/W104A AppA _BLUF_ mutant and for L-tyrosine in solution (inset). Fitting of the sigmoidal curve with a Boltzmann equation reveals a pK = 8.0 for the two tyrosine residues present in AppA_BLUF_ and a pK = 10.5 for L-tyrosine in solution (inset).

FRET efficiency between W104 and FAD (Fig. 2A) has been obtained by two methods: measuring the fluorescence lifetime of the donor (7AW in position 104) in the absence and in the presence of the acceptor (FAD) and by using the method of acceptor enhancement. In this latter case the increase of the acceptor fluorescence intensity is monitored when the donor is present: if the donor transfers energy to the acceptor, enhancement of the fluorescence intensity of the acceptor is observed (see Supplementary Information).

### The 7-aza-Trp analogue (7AW) enables FRET measurements between residue 104 and flavin in the presence of tyrosines

Besides W104, AppA_BLUF_ contains one more tryptophan residue, W64. To eliminate a FRET contribution arising from W64, we have used the W64F mutant in our studies. In addition, to avoid fluorescence emission from the two tyrosine residues (Y21 and Y56) in AppA_BLUF_, we used 295 nm as the excitation wavelength, where tyrosines do not absorb (Fig. S1A). However, the emission spectra of W64F (Fig. 2B) and of the tryptophanless mutant W64F/W104A (Fig. 2D) display a substantial fluorescence emission around 345 nm, suggesting the existence of a fluorophore (other than a tryptophan residue) and hence the presence of another component that may contribute to FRET in addition to W104. The 345 nm emission component is shown below to arise from tyrosinates.

Tyrosine residues are well known to deprotonate at high pH to form tyrosinates (Fig. 2C) which have very similar fluorescent features to tryptophan. In particular, an increase of the pH results in an increase of the absorption at 295 nm with concomitant decrease at 270 nm and a fluorescence emission shift from 303 nm to 340 nm^36,37^. This generally occurs at pH > 10.5 which is the p*K*_a_ of free tyrosine^38^. However, as it has been reported for a series of proteins^39–42^ tyrosine residues can have lower pKa values which results in the formation of tyrosinates and hence fluorescence emission appears around 340 nm, even at pH < 10.5. For example, tyrosinate fluorescence was observed from Y99 in calmodulin from bovine testes and the pKa of this tyrosine residue was shown to be as low as 7.0^41^. Tyrosinate fluorescence was also observed in photoactive yellow protein (PYP) where the pKa changes substantially, depending on whether the tyrosine is solvent exposed, buried or hydrogen bonded^43^, and in chicken ovomucoid where the pKa changes upon pressure dependent unfolding resulting in an increase of the fluorescence emission at 340 nm^37^.

**Figure 3:**
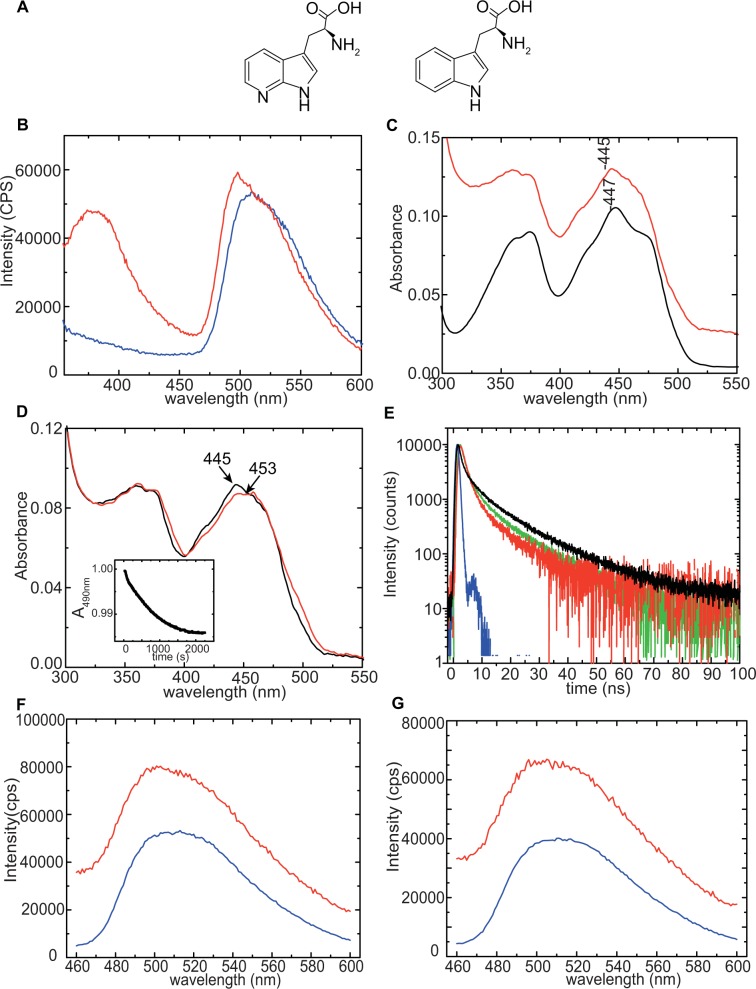
(**A**) Chemical structures showing 7AW (left) and canonical tryptophan (right). (**B**) Emission spectra of W64F (blue line) and 7aza-W104/W64F AppA_BLUF_ mutant (red line) (λ_exc_ = 310 nm). W64F does not show fluorescence emission with 310 nm excitation; around 350 nm the spectrum shows remains of the Raman peak (not shown on this scale). (**C**) Absorption spectra of W64F (black line) and 7aza-W104/W64F AppA_BLUF_ (red line). Spectra are vertically translated for visualization purposes. (**D**) Absorption spectra of 7aza-W104/W64F AppA_BLUF_ in the dark- (black line) and light-adapted (red line) states showing the characteristic red-shift. The inset shows the recovery kinetics of the dark-adapted state monitored at 490 nm after illumination at 385 nm. (**E**) Fluorescence decay of the 7aza-W104/W64F AppA_BLUF_ in the dark (green) - and light-adapted (red) states after excitation at 321 nm, (λ_em_ = 380 nm) in the presence of FAD and of the 7aza-W104/W64F AppA_BLUF_ in the deflavinated, donor-only (black) state after excitation at 321 nm (λ_em_ = 380 nm). The instrument response function is shown in blue. (**F**) Emission spectra of 7aza-W104/W64F AppA_BLUF_ in the dark-adapted state (**G**) Emission spectra of 7aza-W104/W64F AppA_BLUF_ in the light-adapted state. The acceptor only spectra (blue) are calculated as explained in the Supplementary Information. Excitation was set at 310 nm. A significant fluorescence enhancement in the light-adapted state (red) is observed for 7aza-W104/W64F AppA_BLUF_ due to FRET transfer from 7AW to the flavin. The fluorescent enhancement is lesser in the dark-adapted state (**F**).

To test whether tyrosinates are also formed in the AppA W64F/W104A mutant at pH 8.0, we performed pH dependent measurements of the absorbance at 295 nm and determined a pKa value of 8.0 (Fig. 2e). This finding suggests that at pH  8.0, half of the tyrosine residues exists as tyrosinates giving rise to the strong fluorescence emission at ~345 nm. This will necessarily result in a distorted picture for any fluorescence spectroscopy measurement where tryptophan fluorescence is monitored. To eliminate any contribution from tyrosinates, we used 7AW (Fig. 3A) and exploited its spectroscopic properties.

7AW (Fig. 3A) was first incorporated in bacterial proteins 50 years ago^44^. It is an ideal non-invasive *in situ* probe of the structure and dynamics of proteins, as it introduces minimal structural and functional modifications to the protein and in addition has favourable spectral properties compared to canonical Trp and other Trp analogues^45,46^. In particular, the absorption and fluorescence maxima of 7AW are red-shifted by 10 nm and 50 nm, respectively compared to that of canonical Trp (Fig. S1A,B and^45^). This red shift in the absorbance allows us to selectively excite the tryptophan analogue 7AW, using λ_exc_ = 310 nm (canonical Trp has no absorbance at 310 nm) and to avoid the tyrosinates’ absorbance at 295 nm.

Using tryptophan auxotroph cell lines (as described in the Methods section), we incorporated the 7AW analogue in the place of W104 in the W64F mutant of AppA_BLUF_. The successful incorporation of the analogue in W64F AppA_BLUF_ was confirmed by the characteristic emission band at 380 nm (λ_exc_ = 310 nm) which is absent in the W64F mutant (Fig. 3B). The structural modification to the protein, due to the more hydrophilic character of the side chain of 7AW and potential new contacts by means of hydrogen bonds compared to canonical Trp is minimal, as reflected in (1) the flavin absorption spectrum which is similar to that of W64F (Fig. 3C) (2) the characteristic red shift of the isoalloxazine absorption maximum in the light-adapted state is similar to the corresponding shift observed in the W64F mutant (Figs. 3D and S2A) and (3) the identical dark state recovery rate (~14 min) compared to that of W64F (Fig. 3D inset.) The hydrogen bonding environment due to the extra N- atom of the 7aza-W104/W64F AppA_BLUF_ mutant is expected to be different between the wild-type and the 7aza analogue, resulting in an electronically modified structure of the flavin which is reflected as a small shift of the maximum and change in its vibronic structure (Fig. 3D). In the light-adapted state, W104 points towards the flavin. As explained above, the extra N atom of the indole ring of the tryptophan results in a different hydrogen-bonding environment compared to the wild type, leading to different atom displacements in the excited state and hence to different shifts of the maximum for the S0 to S1 transition.

### FRET: Fluorescence lifetime measurements of 7aza-W104/W64F AppA_BLUF_

To determine the FRET efficiency (*E*) between 7AW and FAD we performed time correlated single photon counting (TCSPC) measurements on the tryptophan in the case of the apoprotein and on the protein in the dark-adapted and light-adapted states (Fig. 3E). FRET efficiency was calculated using equation:

2 $$E=1-\frac{{\tau }_{DA}}{{\tau }_{D}},$$

where ${\tau }_{DA}$ is the fluorescence lifetime of the donor (7AW) in the presence of the acceptor (FAD) and ${\tau }_{D}\,$is the fluorescence lifetime of the donor alone. In all three cases the decay of the excited tryptophan analogue is described best using three lifetime components (Table 1) as previously reported for canonical tryptophan. Two of the fluorescence lifetimes of canonical tryptophan (0.2–0.9 ns and 1.2–3.6 ns) are inherent to the tryptophan structure itself independent of the surrounding environment whereas the third lifetime (3.7–9.2 ns) is generated by interactions between the tryptophan and neighbouring residues^47^. In the case of 7AW, the most striking difference compared to the canonical tryptophan is that the third lifetime component is longer (ca. 14–16 ns), whereas the other two components are observed in the same range as for tryptophan. The lifetime of the tryptophan analogue is shorter when FAD is present, suggesting the existence of Förster-type energy transfer. Calculating the FRET efficiency using the average lifetimes we obtained 23% efficiency in the dark-adapted case and 40% in the light-adapted case (see Supplementary information). An estimate of the average distance between the tryptophan analogue and the flavin can be obtained by

3 $$R={R}_{0}^{6}\sqrt{\frac{1-E}{E}}.$$

**Table 1:** Fluorescence lifetimes (ns) of 7AW and corresponding amplitudes in the 7aza-W104/W64F mutant (no FAD present), and the 7aza-W104/W64Fmutant (FAD present) in the dark- and light-adapted states.

7azaTrp-W64F/W104	τ_1_	α_1_	τ_2_	α_2_	τ_3_	α_3_	τ_average_	reference
apoprotein	0.4	0.2	4.1	0.3	16.2	0.4	8.7	this work
holoprotein dark	1.1	0.4	4.4	0.3	16	0.3	6.8	this work
holoprotein light	1.3	0.4	3.5	0.3	14.7	0.2	5.2	this work
canonical Trp	0.2–0.9		1.2–3.6		3.7–9.2			^47^

In the case of 7AW and FAD, *R*_0_ is 16.8 Å using the assumption that the tryptophan can freely rotate thus the value of the orientation factor (see SI) *κ*^2^ = 2/3. In support of free rotation of the tryptophan (*κ*^2^ = 2/3) the anisotropy decay of the dark-adapted state (see next section) is substantially faster when compared to the light-adapted state. Hence in the dark-adapted state, the distance between 7AW and the flavin is estimated to be 20.5 Å (see Supplementary Information). The slower rotational correlation time observed in the case of the light-adapted state of the protein (see below) suggests that the tryptophan is not able to freely rotate as it is hydrogen-bonded in the Trp_*in*_ position. This locked position imposes a strong constraint on the orientation of the transition dipole of W104, implying that *κ*^2^ = 2/3 used for the freely rotating dipoles cannot be used. From the crystal structure (pdb:1yrx) we can observe that the orientation of the transition dipole moments of the flavin and W104 are close to perpendicular (see Fig. S6) thus *κ*^2^ is expected to be close to zero, the theoretical value for perpendicular orientation; we have actually calculated *κ*^2^ = 0.02 (see Supplementary Information). In this case, the value of *R*_0_ is modified to 8.9 Å, and the distance calculated between tryptophan and flavin in the light-adapted state is 9.5 Å.

### FRET: acceptor enhancement method

FRET efficiency between 7AW104 and the flavin can also be calculated by measuring the intensity loss of the emission of the tryptophan or the intensity enhancement of the emission of the flavin due to the resonance energy transfer. In the latter case, known as the acceptor enhancement approach^31,32^, the FRET efficiency is calculated by Eq. (4)

4 $$E=\frac{{\varepsilon }_{A}({\lambda }_{D}^{ex})}{{\varepsilon }_{D}({\lambda }_{D}^{ex})}[\frac{{I}_{AD}({\lambda }_{A}^{em})}{{I}_{A}({\lambda }_{A}^{em})}-1]$$

where ${\varepsilon }_{A}({\lambda }_{D}^{ex})$ and ${\varepsilon }_{D}({\lambda }_{D}^{ex})$ are the extinction coefficients of the acceptor and donor at the donor excitation wavelength $({\lambda }_{D}^{ex})$, and they were calculated using the absorption spectrum of FAD (Fig. S4). ${I}_{AD}({\lambda }_{A}^{em})$ and ${I}_{A}({\lambda }_{A}^{em})$ are the acceptor intensity in the presence and the absence of the donor, respectively.

The enhancement of the fluorescence intensity due to the FRET between tryptophan and flavin was measured in the case of the 7aza-W104/W64F mutant in order to avoid the fluorescence from the tyrosinates as previously discussed.

Figure 3F,G show the emission spectra of the 7aza-W104/W64F mutant in the dark and light states, respectively using λ_exc_ = 310 nm. Inspection of the data reveals a significant increase of the emission of FAD in the presence of the donor both in the dark-adapted and light-adapted states indicating efficient FRET from 7AW to FAD. This fluorescence enhancement is even more pronounced in the light-adapted state. We calculate a FRET efficiency of *E*_dark_ = 42% and *E*_light_ = 59%, which corresponds to a distance *R*_dark_ = 17.7 Å and *R*_light_ = 8.3 Å for the dark- and light-adapted states, respectively^8,48^.

### Comparison of the FRET obtained distances with those from crystal structures

The estimated distances between W104 and FAD obtained from lifetime measurements and the acceptor enhancement method are in relatively good agreement for the dark-adapted state (20.5 Å and 17.7 Å, respectively).

The estimated distances from our fluorescence measurements are also in good agreement with the distances obtained from the crystal structures of AppA_BLUF_ (pdb: 2iyg, 2iyi) and AppA full-length without the Cys-rich region (pdb:4hh0, 4hh1) (Table 2) where W104 has been reported to be located on the surface/distal to FAD (Trp_*out*_ conformation). However, they significantly deviate from those in the first solved crystal structure of AppA_BLUF_ (pdb:1yrx, 5.9 Å) and the NMR solution structure (pdb:2bun, 6.2 Å) for which W104 exhibits the Trp_*in*_ conformation (close to FAD). It should be noted that local differences between the C20SAppA(1–124) dark structure (pdb:2iyg) and the AppA17–133 (pdb:1yrx) have been earlier attributed to the presence of bound detergent molecules^20^, and hence considered for the structure of AppA1–124C20S to represent better AppA_BLUF_ in solution. The significantly (9.4–11 Å) shorter distance between W104 and FAD for the light-adapted state from FRET and fluorescence lifetime measurements suggest that in the light-adapted state W104 adopts a Trp_*in*_ conformation. It should be mentioned that such a difference was not observed between the dark-adapted and light-adapted states of the crystal structures of C20S-AppA(1–124)^20^ (Table 1) potentially originating from constraints in the movement of the indole ring of W104 inside the crystal. This is also reflected in the smaller light-induced red shift in the crystal (5–6 nm) compared to that in the solution (14 nm) suggesting the formation of a preceding intermediate of the full signalling light-adapted state^20^. Interestingly, no significant conformational changes have been observed in the corresponding tryptophan residue (W90) in the photoactivated state of the OaPAC BLUF protein despite the ~10 nm light-induced red-shift observed in the crystal^49^.

**Table 2:** Comparison of the distance between C7 of the indole ring of Trp (W104 in AppA_BLUF_) and N3 of the isoalloxazine ring of flavin from the various crystal structures available for AppA_BLUF_, AppA full-length domains and other BLUF domains.

Protein	pdb/FRET	Distance (Å)	references
AppA_BLUF_ (17–133)	1yrx	5.9	^50^
AppA_BLUF_ (5–125)	2bun	6.2	^28^
C20S AppA_BLUF_ (1–124)	2iyg (dark)	16.0	^20^
C20S AppA_BLUF_ (1–124)	2iyi (light)	16.2	^20^
C20S AppA Δ399	4hh0	15.7	^50^
wt AppA Δ399	4hh1	15.7	^50^
TePixD_BLUF_ (2–143)	1x0p	16.1	^21^
Slr1694_BLUF_ (2–140)	2hfn	15.7, 5.7*	^22^
OAPAC (1–366)	4yus	16.6	^24^
BrlB_BLUF_ (1–140)	2byc	16.0	^23^
7azaW104W64F AppA (dark)	FRET:FL	20.5	this work
7azaW104W64F AppA (light)	FRET:FL	9.5	this work
7azaW104W64F AppA (dark)	FRET: AEM	17.7	this work
7azaW104W64F AppA (light)	FRET: AEM	8.3	this work

An average distance is provided for those structures that contain more than one subunit. *In Slr1694_BLUF_ (2–140), a shorter distance (5.7 Å) is observed in one of the ten subunits. FRET: FL refers to our FRET measurements using fluorescent lifetimes and FRET: AEM to FRET measurements using the acceptor enhancement method.

### 7-aza W104 has restricted movement in the light-adapted state as revealed by fluorescence anisotropy decay measurements

To probe the conformational dynamics of W104 and steric restrictions, we have applied time-resolved fluorescence anisotropy decay measurements, which have been used for decades to characterize protein dynamics. The relaxation of anisotropy induced by a polarized excitation pulse reflects both the dynamics of the fluorophore itself and the protein or segment to which it is attached to. Time dependent anisotropy *r*(*t*) can be fitted as a sum of exponentials^32^,

5 $$r(t)={\sum }_{i}{r}_{0i}\exp \,(-\frac{t}{{\theta }_{i}}),$$

where *θ*_*i*_ values are the rotational correlation times, and *r*_*0i*_ are the limiting anisotropies in the absence of rotational diffusion. In principle, fast rotational correlation times (~1 ns) suggest a fast movement of the fluorophore whereas longer correlation times (tens of nanoseconds) may reflect a slower rotation of the fluorophore or the entire protein complex^32^.

**Figure 4:**
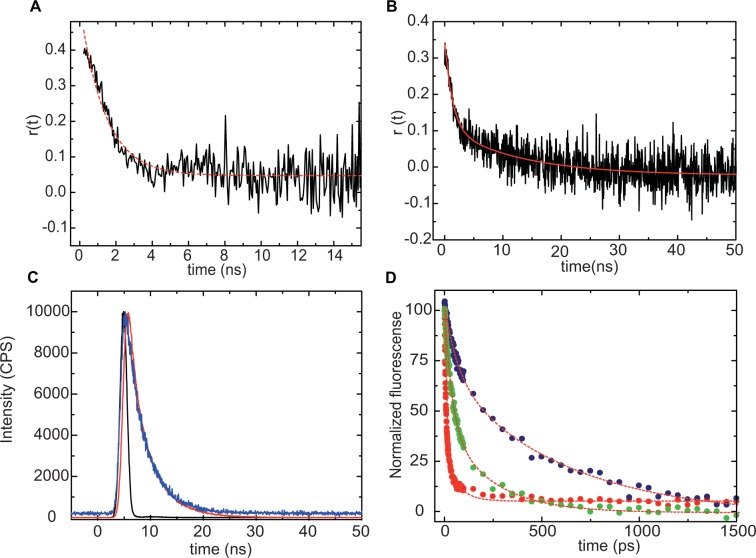
Decay of the fluorescence anisotropy of 7aza-W104/W64F AppA_BLUF_ in the dark-(**A**) and light- adapted states (**B**). (**C**) Fluorescence decay of the Y21F/Y56F/W64F/W104F AppA_BLUF_ (blue) and free FMN (red) after excitation at 455 nm, (λ_em_ = 520 nm). The instrument response function (~ 1 ns) is shown in black. (**D**) Kinetics at the emission maximum (λ_em_ = 520 nm) after excitation at 390 nm for AppA W64F dark-adapted state (navy), light-adapted state (red) and Y21F/Y56F/W64F AppA BLUF (green). The instrument response function was ~ 1 ps.

Figure 4A shows the decay of the fluorescence anisotropy of the 7aza-W104/W64F AppA_BLUF_ mutant in the dark- and light-adapted states. Single exponential fitting reveals a decay constant for the dark-adapted state, τ_*dark*_ = 1.5 ± 0.06 ns and for the light-adapted state, τ_*light*_ = 11.1 ± 0.5 ns. The short rotational correlation time in the dark-adapted state reflects a less restricted rotation of the tryptophan residue, and it is close to the rotational correlation time of L-tryptophan in buffer (*θ* = ~0.6 ns) implying an almost free rotation of the indole moiety.

The observed 11 ns long rotational correlation time in the light adapted state corresponds to the rotation of the whole protein and agrees well with the value expected based on the empirical formula proposed by Visser^51^: $\Phi =3.84\,{10}^{-4}{M}_{r}$, where Φ is the rotational correlation time in ns and *M*_*r*_ is the molecular weight of the protein in Da. This observation suggests that upon blue light absorption, the W104 tryptophan residue moves closer to the flavin and gets hydrogen bonded to an adjacent amino acid residue, most probably Q63.

Fluorescence anisotropy decay measurements were performed in the Y21F/Y56F/W64F AppA mutant as well, in which case the protein is locked in a state which is very similar to the light adapted. The rotational correlation time was calculated to be ~10 ns (see Fig. S5), hence very similar to the one observed in the light-adapted state of the 7aza-W104/W64F mutant. That finding suggests that the tryptophan at 104 position is rigidly bound to the protein, in the triple mutant.

These results are in line with the findings from our FRET measurements: W104 in AppA_BLUF_ is characterized by flexible dynamics in the dark- adapted state whereas in the light-adapted state, it is present in a more restricted environment, probably due to a stronger hydrogen bond network around the flavin molecule, originating from the nitrogen atoms of the tryptophan that are expected to form additional hydrogen bonds with Q63 and O14 of the flavin. It should be pointed out that the flexibility of W104 in the dark-adapted state is a novel finding that hasn’t been observed in previous studies which have favoured a position of the W104 close to the flavin in a Trp_*in*_ conformation^18,19,29^.

### Fluorescence lifetimes of the flavin in the dark- and light-adapted state

Ultrafast electron transfer from the neighbouring aromatic amino acid residues (Trp and Tyr) to the electronically excited flavin chromophore has been observed in many flavoproteins (including glucose oxidase, flavodoxin, riboflavin binding protein, photolyase/cryptochromes and ThyX)^52–56^. This photoinduced electron transfer (PET) results in substantial shortening of the fluorescence lifetime of the flavin. Measurements of the fluorescence lifetime of the flavin can therefore provide significant information on the flavin environment. Here, we applied TCSPC and Kerr-gated fluorescence spectroscopy^57^ to determine the fluorescence lifetimes of the flavin in the dark- and light-adapted state in order to obtain further information on the position of W104 during photoactivation, and its influence on the quenching mechanism.

As expected, in the AppA_BLUF_ mutant where all four tyrosine and tryptophan residues have been mutated (Y21F/Y56F/W64F/W104F) and hence all possible electron donors are removed, the fluorescence lifetime of the flavin was 3.8 ns (Fig. 4B). This is in the range expected for the fluorescence lifetime of the flavin in solution ~3–5 ns: the fluorescence lifetime (the longer component) of FAD is around 3 ns^58^, free FMN in water is around 4.8 ns, lumiflavin is 5.2 ns^59,60^. As expected from the lower quantum yield of FAD fluorescence in AppA W64F (8.5% and 4.8% in dark- and light-adapted state respectively) a substantially faster fluorescence decay with respect to that of the quadruple mutant was observed both in the dark-and light-adapted case (Fig. 4C and Table 3).

**Table 3:** Fluorescence decay constants of the flavin and corresponding amplitudes in various AppA_BLUF_ mutants.

Protein	A1	T1(ps)	A2	T2(ps)	R1 (Å)	R2 (Å)
W64F dark	0.7	**500**	0.3	70	9.5	8.03
W64F light	0.13	230	0.87	**11**	8.9	6.7
Y21F/Y56F/W64F	0.43	200	0.57	**36**	8.8	7.6

Estimated distances between the flavin and W104 based on electron transfer calculations. The major contribution is shown in bold.

Transient picosecond fluorescence spectra (Fig. S3A–C) and kinetics at the emission maximum (λ_max_ = 520 nm) (Fig. 4D) are shown for W64F AppA (dark- and light-adapted states) and Y21F/Y56F/W64F AppA. Global analysis reveals a biphasic fluorescence decay for the flavin. (Table 3). In the dark-adapted state of the W104/W64F, there is a dominant phase with a decay constant τ_*dark*_ = 500 ps that is around fifty times slower than the dominant phase of the light-adapted state, τ_*light*_ = 11 ps. The faster decay in the light-adapted state, suggests that the excited state of the flavin is quenched by electron transfer from close-by aromatic residues that should be positioned closer to the flavin compared to their position in the dark-adapted state.

We measured the fluorescence lifetime of the flavin in the Y21F/Y56F/W64F AppA mutant where all the potential electron donors were removed except for W104. In this case we also observed a significantly (~4–5 times) shortened relaxation time compared to the time constant measured in the dark-adapted state of W64F AppA. This indicates that the tryptophan in this mutant is closer to the flavin than in dark-adapted state of AppA W64F and it is able to quench efficiently the excited state of the flavin. This result lines up well with the findings from the anisotropy decay measurements (Fig. S5) altogether indicating that W104 is in a rigidly bound position close to the flavin.

We therefore assign the drastic acceleration of the fluorescence decay in the light-adapted state of AppA W64F to the movement of the W104 towards the flavin and its acting as the main quencher in the light-adapted state (see Discussion). Despite the fact that in PixD the respective tyrosine (Y8) is involved in proton coupled electron transfer (PCET) we don’t anticipate that Y21 is an efficient quencher in the case of AppA_BLUF_. We have already demonstrated^9^ the lack of an efficient ET during the forward reaction in AppA_BLUF_ which we attribute to the low pKa of Tyr21. Our previous work^73^ on PixD where we replaced Tyr8 with fluorinated tyrosines, lowering the pKa of the tyrosine (by fluorination) led to an increase of the lifetime of the excited state. The same effect was observed in another BLUF protein (unpublished results) implying that the ability of the respective tyrosine to quench the flavin decreases with low pKa. The possible reason is that despite the higher driving force for ET, tyrosinate formation prevents the PCET which is clearly needed in PixD.

In addition, the emission spectra of the W64F AppA_BLUF_ (Figs. 2B and S3B) lose the characteristic vibrational shoulder at ~500 nm in the light-adapted state. These changes are also accompanied by a decrease in the fluorescence (Fig. 2B) and are indicative of an enhanced electron transfer in the case of the light-adapted state.

Using the simple empirical expression known as the Dutton ruler (Eq. 6)^61^

6 $$\log \,{k}_{ET}=15-0.6R-3.1\frac{{(\varDelta G+\lambda )}^{2}}{\lambda }$$

that relates the electron transfer rate constant (*k*_*ET*_) to the edge-to-edge distance (*R* in Å) between two reaction centres, we roughly estimated the distances between flavin and the tryptophan for the dark-adapted state (*R*_dark_ = 9.5 Å) and the light-adapted state (*R*_light_ = 6.7 Å) (Supplementary information), assuming a barrierless electron transfer (Δ*G* = −*λ*), where Δ*G* is the driving force for the electron transfer between the electron donor (presumably W104 at least for the light-adapted state) and the excited flavin and *λ* is the reorganization energy.

The triple mutant (Y21F/Y56F/W64F) where both tyrosines and W64 tryptophan were removed is photoinactive, no red shift is observed upon blue light illumination. The absorption spectra of this mutant and the Y21F/Y56F/W64F/W104F mutant can be found in the Supplementary Information (Fig S7). Both mutants are very stable even at high concentrations (300–500 μM) and able to retain the flavin despite the multiple mutations. In the case of the Y21F/Y56F/W64F mutant it seems that W104 is locked in a position that is characterized by similar photophysics with the light-adapted state of W64F AppA_BLUF_ as the fluorescence decay of the flavin is very fast. Interestingly, the two decay phases of the flavin have similar contributions indicating that W104 exists in two conformations relative to its proximity to the flavin chromophore. Based on the Dutton ruler, the following estimated distances were calculated, *R*_*in*_ = 7.6 Å (*τ* = 36 ps) and *R*_*out*_ = 8.8 Å (*τ* = 200 ps).

### Transient infrared measurements

**Figure 5:**
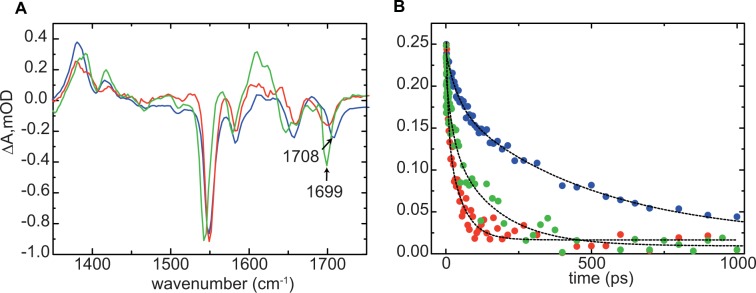
(**A**) TRIR spectra of dark- and light-adapted (blue and red respectively) W64F and the Y21F/Y56F/W64F AppA mutants (green) recorded at 10 ps; (**B**) Kinetics of the excited state of the flavin in the dark- and light-adapted (blue and red respectively) states of W64F AppA_BLUF_ and in the Y21F/Y56F/W64F AppA_BLUF_ (green) observed at 1380 cm^-1^.

To study the protein dynamics in the AppA_BLUF_ domain after blue light absorption we performed transient infrared measurements on the dark- and light-adapted states of AppA_BLUF_ W64F and on the AppA_BLUF_ Y21F/Y56F/W64F mutants (Fig. 5A). Transient infrared spectra recorded at time delays between 1 ps and 2 ns after 450 nm excitation of the flavin show similarities with the spectra observed for the wild type AppA_BLUF_^62,63^. The negative features (bleaches) of the recorded spectra associate with depletion of the ground state population or with photoinduced changes in the vibrational spectrum of the protein, occurring either directly through electronic excitation at *t* = 0 or as a result of subsequent structural dynamics; positive bands are associated with vibrations of the electronically excited state of the flavin, or with modes of the protein which shift as a result of electronic excitation, or of products formed subsequently. In AppA_BLUF_ (and the majority of flavoproteins) four dominant bleaches are observed in the 1300–1800 cm^−1^ region: the bands at 1547 cm^−1^ and 1581 cm^−1^ arise from ring modes of the isoalloxazine ring whereas the ~1650 cm^−1^ and ~1710 cm^−1^ bands are related to the carbonyl stretches C2=O and C4=O of the FAD ground state, respectively, and are sensitive to the H-bond environment^62,64^.

Comparison of the dark- and light adapted spectra of the W64F AppA mutant reveals a ~9 cm^−1^ downshift for the C4=O carbonyl band in the light adapted state, that is similar to the shift observed for the WT AppA^63^ and attributed to the formation of new hydrogen bond to C4=O. That result demonstrates the arrangement of a rigid environment for W104 in the light-adapted state due to the formation of a H-bond to the flavin, and supports our FRET measurements that reveal a change of the location of W104 close to the flavin in that state. It is also in line with our anisotropy decay measurements that demonstrate a slower rotation (~11 ns) for W104 in the light-adapted state, reflecting rotation of W104 together with the whole protein due to the more restricted environment of W104. Similarly, the vibrational mode related to the C4=O carbonyl group is downshifted in the Y21F/Y56F/W64F AppA_BLUF_ compared to the dark-adapted W64F AppA_BLUF_ suggesting a bond strengthening. Figure 5B shows the kinetics observed at 1380 cm^−1^ which reflect the decay of the excited state of the flavin^9^. The kinetics in Table 4 are in line with our findings from the picosecond fluorescence lifetime measurements for the flavin: the decay of the excited state is slower in the dark-adapted state and faster in the light-adapted state reflecting the efficient quenching of the excited state in the latter case. The kinetics for the Y21F/Y56F/W64F reflect also a significantly faster relaxation compared to the AppA_BLUF_ W64F dark-adapted state which allows us to conclude that W104 (the only possible electron donor in this case) is in a rigidly bound and closer position to the flavin compared to the dark-adapted state.

**Table 4:** Decay time constants at 1380 cm^−1^.

	τ_1_ (ps)	α_1_	τ_2_ (ps)	α_2_
Dark W64F	41	0.27	482	0.73
Light W64F	23	0.86	221	0.14
Y21F/Y56F/W64F	23	0.36	160	0.64

## Discussion

W104, a residue present in the majority of BLUF containing photoreceptors [AppA, Slr1694, OaPAC, TePxD, BlrB]^49^ is of special interest because of the inconsistencies reported concerning its conformation in the dark- adapted states, based on crystal structures and its potential role in the long-range signalling. It is well known that X-ray crystal structures of proteins capture only those conformations that fit into the crystal lattice, which may or may not be relevant to function.

Earlier spectroscopic studies^18,29^ have favoured the Trp_in_ conformation for the dark-adapted state based on indirect evidence for a buried and hydrophobic environment around the tryptophan. A fluorescence spectroscopy approach was used by Dragnea *et al*.^18^ where they used acrylamide quenching to characterize the dynamics of the respective tryptophan. Acrylamide quenching was proved to be a useful method to obtain information about the tryptophan accessibility. The quenching of the fluorescence of a buried tryptophan is less efficient than the quenching of a solvent exposed one. In the previous experiments though, the results of acrylamide quenching were influenced by the unexpected appearance of tyrosinate fluorescence, revealed here. Incorporating the unnatural amino acid 7AW we were able to avoid the fluorescence of tyrosinate, in this way our work provides unequivocal evidence, as it relies on a novel approach and the application of advanced fluorescence techniques to address the orientation of W104 in the BLUF functional states.

Fluorescence spectroscopy methods – namely fluorescence anisotropy decay and Förster-type energy transfer– were used to both characterize the mobility and position of W104 tryptophan during photoactivation, and its distance from flavin in dark- and light-adapted states. In the case of the FRET experiments we monitored the changes of the fluorescence intensity of the acceptor as there was no possibility to measure the FRET efficiencies from the donor side; removing the flavin would have led to a photoinactive protein. In order to measure the FRET efficiency using the acceptor enhancement method we first made the W64F/W104A mutant which was anticipated to have a fluorescence intensity arising only from the acceptor following excitation at 295 nm. However, an unexpectedly strong fluorescence signal around 340 nm was observed, despite the lack of the tryptophans, pointing to substantial fluorescence from the tyrosinate form of tyrosines in AppA_BLUF_. In order to eliminate this fluorescence emission, which overlaps with the fluorescence coming from the tryptophans, we used the 7AW tryptophan analogue which has shifted absorption and fluorescence spectra compared to the canonical one.

Substitution of W104 with the unnatural amino acid residue 7AW resulted in a tailor made BLUF protein with useful spectral properties that allowed us to obtain clear and distinct fluorescent signals, making possible measurement of the fluorescent anisotropy decay of 7AW in the 104 position of AppA_BLUF_ in the dark- and light-adapted state of the protein. The outcome of the fluorescence anisotropy decay measurements resulted in an unexpected picture compared to our presumption: the fast anisotropy decay (~1 ns) of 7AW in the dark- adapted state reflects the nearly free rotation of the tryptophan suggesting that it is therefore not hydrogen bonded to the flavin and the adjacent glutamine (Q63). In contrast a slow rotation (~11 ns) was observed in the light- adapted state, which shows that the tryptophan is rotating together with the protein, most probably because it is tightly hydrogen bonded to Q63.

The substitution of W104 by the tryptophan analogue 7AW paved the way for the FRET experiments using the “spectroscopic ruler” to determine the tryptophan-flavin distance in both the dark- and light-adapted states. The estimated distance obtained from the acceptor enhancement method for the dark-adapted state (17.7 Å) is in good agreement with the distance obtained from the crystal structures of the AppA_BLUF_ (16 Å) and full-length protein (15.7 Å) (Table 2). A slightly longer distance (20.5 Å) was obtained from the fluorescence lifetime measurements probably due to the uncertainty in the estimation of the fluorescence lifetime of 7AW in position 104 in the apoprotein. In the absence of the flavin, the protein may adopt a different conformation compared to when flavin is present which may result in a modified fluorescence lifetime as the tryptophan is very sensitive to the protein environment. The FRET measurements for the dark-adapted state point towards a more distant position for the tryptophan compared to the light-adapted state in which the tryptophan is very close (8.3/9.5 Å, Table 2) to FAD. The estimates of 8.3/9.5 Å center-to-center between FAD and 7AW-Trp104 from the FRET experiments are in good agreement with the estimate of 6.7 Å (edge-to-edge) between FAD and W104 from the electron transfer quenching experiments. This assessment also confirms that W104 acts as the predominant electron donor to FAD* (excited FAD) in the light-adapted state.

The change in the orientation and the distance of W104 during the photoactivation was reflected in earlier fluorescence and transient absorption spectroscopy measurements. Streak camera measurements on the dark-adapted AppA_BLUF_ have shown that the fluorescence lifetime of flavin is ~ 600 ps^10^ in line with our measurements (Table 3). This value drops by a factor of ten (Table 3) in the case of the light-adapted state of the protein suggesting that flavin fluorescence is strongly quenched most probably by the nearby tryptophan, hence supporting a different location for the tryptophan residue closer to the flavin molecule.

Results from visible and infrared transient absorption measurements are also consistent with the above measurements: in the light-adapted state a flavin and tryptophan radical was formed, which is not the case for the dark-adapted state^9,58^ (in agreement with our assessment that W104 is not a main quencher in the dark-adapted state). These observations can be explained by the simple mechanistic scheme that in the light-adapted state efficient electron transfer takes place by the closely positioned tryptophan that can provide an electron to the excited flavin, quench it and form the flavin radical. As discussed above, the distances obtained from the electron transfer calculations are in good agreement with those obtained from the FRET measurements supporting an electron transfer from the tryptophan residue. This finding is underlined by the strong quenching of the excited state observed in the Y21F/Y56F/W64F mutant where the only possible electron donor is W104 and it is in a close position (7.6 Å) to the flavin. Fluorescence anisotropy decay measurements performed on this mutant show a similar rotational correlation time (see Fig. S5) to that measured in the light-adapted case, indicating that the tryptophan in this case is locked – most probably via hydrogen bonding –in a light-like state.

The TRIR data measured on the light-adapted W64F and Y21FY56FW64F mutant show a downshift of frequency of C4=O carbonyl which can be explained based on the model suggested by Iwata *et al*.^65^ (Model 7) which assumes a hydrogen bond formed between the enol form of Q63 and W104.

All these findings agree with recent calculations^27^ which show that the close position of W104 is favourable for electron transfer from tryptophan to flavin and this pathway is dominating when the transfer is from Y21 to the flavin is repressed by the lack of the conducive hydrogen-bonding network. Overall our study addresses previous discrepancies on the subtle conformational differences of W104 that characterize the dark and signalling states in BLUF containing photoreceptor proteins, and provides significant information that allow us to re-examine the existing mechanistic models of photoactivation^63^. The hierarchical relaxation pathway after flavin photoexcitation in the AppA_BLUF_ domain occurs on the pico- to nanosecond time scale for the flavin localized dynamics while subsequent protein structural reorganization is observed over microseconds^66^. Rearrangement of the hydrogen bond network around the flavin is believed to be associated with conformational changes involving the β5 strand^18,28^. The movement of the W104 residue which resides on the β5-strand is therefore not unexpected and can result in further structural changes in the C-terminal α-helices affecting the interaction of the protein with downstream components. Understanding the dynamic behaviour of photoreceptors has a major impact on the field of optogenetics as it can help to control gene expression and engineered biosynthetic pathways.

## Conclusions

A wide range of fluorescence techniques, time-resolved infrared spectroscopy and unnatural amino acid incorporation have been used to probe the functional dynamics of W104 during the photoactivation process in AppA_BLUF_. Fluorescence resonance transfer (FRET) measurements and replacement of W104 with the unnatural amino acid 7AW have provided quantitative information on the position of W104 in the dark- and light-adapted states. In the light-adapted state, W104 is in a restricted environment with an enhanced H-bond network compared to that in the dark-adapted state, as revealed by the long rotational correlation time (~11 ns) for W104 from fluorescence anisotropy decay measurements and the downshift of the C4=O carbonyl band from infrared measurements. In addition, in the light-adapted state the short fluorescence lifetime of the flavin suggests that efficient electron transfer takes place to the excited flavin from the closely placed W104. Overall, our study addresses previous discrepancies on the position of W104 during the photocycle of AppA and supports a conformation of W104 close to the flavin in the light-adapted state whereas in the dark-adapted state W104 is present in a less restricted environment pointing away from the flavin.

## Methods

### Materials

Glycogen (Type III: from rabbit liver) was purchased from Sigma-Aldrich. 7AW was purchased from Aldrich.

### Expression and purification of W64F, W64F/W104A, 7aza-W104/W64F, Y21F/Y56F/W64F and Y21F/Y56F/W64F/W104F AppA_BLUF_ proteins

The BLUF domain of AppA (AppA_BLUF_: residues 5–125) was expressed in BL21(DE3) *Escherichia coli* cells. Protein expression and purification were performed in the dark as described previously^63^. The constructs for the site-directed mutants W64F, W64F/W104A and Y21F/Y56F/W64F were generated by PCR amplification (overlap extension method) from wild-type pET15b_AppA_BLUF_ using the NdeI and BamHI restriction enzymes. The primers used are described in Supplementary Table 1. The DNA sequence for expressing the Y21F/Y56F/W64F/W104F AppA_BLUF_ mutant was purchased from Thermo Fisher Scientific. All constructs were verified by DNA sequencing.

The 7aza-W104/W64F construct was expressed in a Tryptophan auxotroph M5219 *Escherichia coli* strain, purchased from the Belgian Coordinated Collection of Micro-Organisms (http://bccm.belspo.be/). The W64F AppA_BLUF_ contained construct was transformed by heat shock into M5219 *E. coli* strain. A single colony was inoculated into 25 mL Luria Broth (LB) media incubated overnight at 200 rpm and 37 °C. The overnight culture was used to inoculate 1 L M9 medium in a 4 L flask and cells were allowed to grow at 30 ^o^C until OD_600nm_ reached ~0.6. At this point, the cells were washed three times with washing buffer (M9 mineral salts solution and 20% glucose) to remove all Trp from the medium harvested, resuspended in 1 L M9 media and cultured for additional 30 minutes at 30 ^o^C to ensure full Trp depletion^66^. 7AW was then added and incubated for 15 minutes to allow the cells to uptake it after which the temperature was decreased to 18 ^o^C for 30 min followed by addition of 0.8 mM isopropyl β-D-1-thiogalactopyranoside (IPTG) to induce protein expression. Cells were harvested, lysed and purified using Ni-NTA affinity resin as previously described^63^. The apoprotein is produced during protein expression and was isolated by size exclusion chromatography.

### Steady state optical and fluorescence measurements

Absorption spectra were measured on a Perkin Elmer Lambda XLS + and Jasco V-660 spectrophotometer. Fluorescence emission spectra were obtained with a Horiba Jobin Yvon Fluorolog spectrofluorometer using three excitation wavelengths (λ_exc_ = 295 nm, λ_exc_ = 350 nm, λ_exc_ = 455 nm). The applied slit width was set at 5 nm for both the excitation side and the emission side. Unless otherwise indicated all fluorescence spectra were measured in the dark at 22 ^o^C in a 10 mm × 1 mm quartz cuvette.

### Fluorescence lifetime and anisotropy decay measurements

#### Fluorescence anisotropy decay measurements

Fluorescence anisotropy (*r*) measures the depolarization of the fluorescence emission of a fluorophore due to energy transfer to another molecule with a different orientation or molecular rotation caused by Brownian motion. As molecular motion depends on the size of the fluorophore and on local environment factors such as viscosity and molecular confinement, fluorescence anisotropy measurements can provide useful information on the molecular size and the mobility of the fluorophore^32^. In the case of continuous excitation, the steady-state anisotropy $\bar{r}$ is given by Eq. (7)

7 $$\bar{r}=\frac{{I}_{\parallel }-{I}_{\perp }}{{I}_{\parallel }+2{I}_{\perp }}$$

where *I*_*||*_ and *I*_*⊥*_ are the intensities measured with a linear polarizer for emission parallel and perpendicular, respectively, to the electric vector of linearly polarized incident light.

In a fluid environment, the initial uniaxial distribution of photo-selected molecules is usually randomized by rotational diffusion, leading to depolarization of fluorescence. In the case of free rotation, the fluorescence anisotropy decays to zero, while for hindered rotation the anisotropy reaches a time-independent value at long times, *r*_∞_, called the *residual anisotropy*. The analysis of the temporal decay of the anisotropy yields direct information on the orientational dynamics, which depends on the size and shape of the rotating species and on the fluidity of its microenvironment^31,32^.

For an excited species in a single type of isotropic environment, *r*(*t*) is, in general, given by a linear combination of exponentially decaying functions

8 $$r(t)={\sum }_{i}{\rm{\beta }}i\,\exp \,(-\frac{t}{{\theta }_{i}})$$

where θ_*i*_ are the rotational correlation times. Obviously, the sum of the factors β_i_ yields the initial emission anisotropy *r*_0_.

#### Nanosecond time-resolved fluorescence measurements

Time-resolved fluorescence measurements in the nanosecond time range were performed on a Horiba Jobin Yvon Nanolog FL3-2Ihr spectrofluorometer operating in the time-correlated single photon counting (TCSPC) mode and coupled to a R928P Hamamatsu photomultiplier. The applied detector voltage was set at 950 V. The measurement range of the TCSPC system was 200 ns, the repetition rate of the excitation pulses was 1 MHz, and the sync delay was 50 ns. Decay curves were collected in 4096 channels of a multi-channel analyser using a channel width of 55 ps. For the deconvolution procedure, the dynamic instrument response function (IRF) was determined using a freshly made solution of glycogen in water (half-bandwidth ~1 ns).

To measure the fluorescence lifetimes, the excitation wavelength was set at 321 nm (7aza-tryptophan excitation) and 455 nm (FAD excitation) using pulsed nanoLEDs (Horiba) with pulse duration <1 ns and 1.2 ns, respectively. The fluorescence emission was collected at 380 nm (λ_exc_ = 321 nm) and at 520 nm (λ_exc_ = 455 nm). The appropriate emission wavelength was set by a built-in the system monochromator. All measurements were performed using the Data Station v 2.6 software and the lifetime and anisotropy data were analysed using the Jobin Yvon DAS6 v6.6 and FluoFit softwares.

To generate and maintain the light-adapted state of the BLUF proteins, a Thorlabs M385 L2-C1 UV Led (λ_exc_ = 385 nm) was placed above the sample compartment and covered by a black cloth. The light-adapted state was reached after 10 minutes of illumination. Sample concentration was in the 5–10 µM range. All samples were illuminated continuously during the fluorescence measurements. To eliminate the 385 nm scattered light, an UG11 filter was placed between the sample and the detector. 10 mm × 1 mm quartz cuvettes were used for the measurements. UV-vis absorption spectra were obtained before and after the fluorescence measurements to monitor the integrity of the light-adapted state, using a Thermo Scientific Evolution 600 UV-vis spectrophotometer.

#### Picosecond time-resolved fluorescence measurements

Time-resolved fluorescence experiments in the ps time range were performed using a spectrally resolved Kerr-Gate femtosecond fluorometer. The setup employs a Kerr shutter^67,68^ and allows measuring fluorescence spectra with a resolution of ~100 fs and up to the nanoseconds timescale^57^. The setup was described elsewhere^52,72^ but briefly, the excitation pulse centered at 390 nm is obtained by frequency-doubling, using a BBO crystal, part of the 780 nm pulse from the Ti:sapphire laser/amplifier system (Quantronix Integra-C) operating at 1 kHz. The remaining 780 nm beam is led through a motorized delay-line and focused into the Kerr medium where it spatially overlapped the fluorescence from the sample. The Kerr medium used was CS2 (response function width ~1.2 ps). The sample was flowed through the 1 mm pathlength optical cell using a peristaltic pump. To generate and maintain the light-adapted state the sample reservoir was illuminated using a ThorLabs M450LP1 LED (λ_exc_ = 450 nm). Transient fluorescence spectra were measured on time windows up to 1500 ps for all samples. Global analysis of the time and spectrally resolved data sets in terms of a linear combination of a discrete number of components, each with a distinct exponential rate constant and decay-associated spectrum^69^, was completed using Glotaran^70^.

### Transient infrared spectroscopy

All transient infrared absorption (TRIR) measurements reported here used the 10 kHz ULTRA facility developed at the Central Laser Facility of the Rutherford Appleton Laboratories which are described in detail elsewhere^71^. As described earlier^9,15,71^ ULTRA offers wide tunability in the visible region, a broad bandwidth in the IR probe, sub 100 fs rime resolution and excellent stability. In the present experiments it was used in the visible pump – IR probe geometry. The excitation (pump) wavelength was 450 nm with a pulse energy of a few hundred nJ focussed to a 100 micron spot size. It was checked that the spectra and kinetics were independent of the pump wavelength and pulse energy. IR transmission was measured sequentially for pump-on and pump-off using a 5 kHz mechanical chopper, and the data were processed to provide the TRIR difference spectra.

## Supplementary information

Supplementary information.
