# Supplementary material for: Functional dynamics of a single tryptophan residue in a BLUF protein revealed by fluorescence spectroscopy
Source: Sci Rep. 2020 Feb 6;10:2061. doi: 10.1038/s41598-020-59073-5 (PMC7005313; doi:10.1038/s41598-020-59073-5)
Supplement: Supplementary file 1 — Supplementary information. [file 41598_2020_59073_MOESM1_ESM.pdf]

Supplementary Information

Functional dynamics of a single tryptophan residue in a BLUF protein revealed by fluorescence spectroscopy

Kristof Karadi[1,2], Sofia M. Kapetanaki[1,2], Katalin Raics[1], Ildiko Pecsi[1], Robert Kapronczai[1], Zsuzsana Fekete[1], James Iuliano[3], Jinnette Tolentino Collado[3], Agnieszka Gil[3], Jozsef Orban[1], Miklos Nyitrai[1,2], Greg M. Greetham[4], Marten H. Vos[5], Peter J. Tonge[3], Stephen R. Meech[6], Andras Lukacs[1,2]

[1] Department of Biophysics, Medical School, University of Pécs, 7624 Pécs, Hungary

[2] Szentagothai Research Center, University of Pecs, 7624 Pécs, Hungary

[3] Department of Chemistry, Stony Brook University, Stony Brook, NY, 11794-3400, USA

[4] Central Laser Facility, Harwell Science and Innovation Campus, Didcot, Oxfordshire, UK.

[5] LOB, CNRS, INSERM, Ecole Polytechnique, Institut Polytechnique de Paris, 91128 Palaiseau Cedex, France

[6] School of Chemistry, University of East Anglia, Norwich NR4 7TJ, UK

Calculation of distance R between Trp104 and flavin in the dark- and light-adapted states of 7aza-W64F/W104F.

I. *From fluorescence lifetime measurements of Trp104:*

The FRET efficiency was calculated using equation (1): $E = 1 - \frac{\tau_{DA}}{\tau_D}$ (1),

where τ_{DA} is the fluorescence lifetime of the donor (tryptophan analogue) in the presence of the acceptor (FAD) and τ_D is the fluorescence lifetime of the donor alone. Using the average lifetimes (Table 1), we obtain:

$E_{dark} = 1 - \frac{6.79}{8.73} = 1 - 0.77 = 0.23$, for the dark-adapted state and

$E_{light} = 1 - \frac{5.24}{8.73} = 1 - 0.6 = 0.4$, for the light-adapted state

The average distance between the tryptophan analogue and the flavin can be inferred from the FRET efficiency using equation (2): $E = \frac{1}{1+(\frac{R}{Ro})^6}$ (2), where R is the distance between the donor and the acceptor and R_0 is the Förster radius, the distance between the two fluorophores in the case of 50% transfer efficiency.

R_o is given from equation (3) $Ro = 9.78 * 10^3 [J(\lambda)\kappa^2 n^{-4} \Phi_D]^{1/6}$ Å (3),

where $J(\lambda)$ is the integral of spectral overlap between donor emission and acceptor absorption at λ nm, n is the refractive index typically assumed to be 1.4 for biomolecules[1] and Φ_D is the quantum yield of the donor ($\Phi_{7azaTrp}$ =0.01 and $\Phi_{Trp=}$ 0.13)[2] . κ^2 is the orientation factor that can be calculated from equation (4), $\kappa^2 = (\cos\theta_T - 3\cos\theta_D \cos\theta_A)^2$ (4)

where θ_T is the angle between the transition dipole moments of the donor and the acceptor, and θ_D and θ_A are the angles between these dipole moments and the separation vector. A value of κ^2=2/3=0.66 is assumed for randomized orientation and κ^2=0 for perpendicular orientation. Anisotropy decay of 7AW in dark-adapted state has shown that the tryptophan analogue is able to move freely, therefore we used κ^2=2/3 for calculating R_0 inthis case. Slower rotational correlation time observed in the light adapted case indicated a restricted motion of 7AW, therefore we used the Trp_{in} structure (pdb:1YRX) to calculate κ^2 for the light-adapted state. Based on the structure (pdb:1YRX), the transition dipole moment of the tryptophan forms an θ_A=80° angle with the intersection line of the planes containing the flavin and the tryptophan (Fig. S6). The two planes form a ϕ ~ 63° which results in a κ^2 =0.02. We should note here that there is an uncertainty in the determination of κ^2 as the dihedral angle varies between 63° and 74°.

From equation (2) we obtain, $R = R_o \sqrt[6]{\frac{1-E}{E}}$ (5).

In the dark-adapted state R_0 between 7AW and FAD is 16.8 Å [Förster distance calculator (version 1.0c, UMASS)] using the assumption that the tryptophan can freely rotate thus the value of κ^2 is 2/3. The distance calculated is $R = 16.8 * \sqrt[6]{\frac{1-0.23}{0.23}}$ =20.5 Å for the dark-adapted state.

For the light-adapted state we calculated κ^2= 0.02, thus R_0=8.9 Å and the distance calculated between the tryptophan and the flavin is $R = 8.9 * \sqrt[6]{\frac{1-0.4}{0.4}} = 9.5$ Å.

II. Acceptor enhancement method:

Calculation of distance R between Trp104 and flavin in the dark state of 7aza-W104/W64F.

Using the acceptor enhancement approach[1,3] the FRET efficiency is calculated by equation (2)

$$E = \frac{\varepsilon_A(\lambda_D^{ex})}{\varepsilon_D(\lambda_D^{ex})}\left[\frac{I_{AD}(\lambda_A^{em})}{I_A(\lambda_A^{em})} - 1\right] \quad (2)$$

where $\varepsilon_A(\lambda_D^{ex})$ and $\varepsilon_D(\lambda_D^{ex})$ are the extinction coefficients of the acceptor and donor at the donor excitation wavelength ($\lambda_D^{ex} = 310\ nm$), and $I_{AD}(\lambda_A^{em})$ and $I_A(\lambda_A^{em})$ are the acceptor intensity in the presence and the absence of the donor at the acceptor emission wavelength ($\lambda_A^{em} = 505\ nm$). In particular, $\varepsilon_A(\lambda_D^{ex}) = \varepsilon_{FAD}(310nm) = \varepsilon_{FAD}(450nm) * \left(\frac{OD310nm}{OD450nm}\right) =$ 8500 *0.101=860.32 M^{-1} cm^{-1}. The absorption spectrum of flavin is shown in Figure S4.

$\varepsilon_D(\lambda_D^{ex}) = \varepsilon_{7azaTRP}(310nm) = \varepsilon_{7azaTRP}(max) * \left(\frac{OD310nm}{ODmax}\right) = 1013$ cm^{-1}. The absorption spectrum of 7-aza Trp is shown in Figure S1a.

The molar extinction coefficient of FAD is ε_{FAD}=8500 $M^{-1}cm^{-1}$ at 450 nm[4],

the molar extinction coefficient of tryptophan is ε_{TRP}=6000 $M^{-1}cm^{-1}$ at 290 nm[5].

For the 7aza-W104/W64F mutant, $I_{AD}(\lambda_A^{em})$ is 78940 cps in the dark state (Figure 3f).

The emission intensity of the 7aza-W104/W64F mutant in the absence of the donor [$I_A(\lambda_A^{em})$] was calculated using the ratio with $\frac{I_{350nm}(\lambda_{FAD}^{505nm})}{I_{310nm}(\lambda_{FAD}^{505nm})} = 4.71$ measured in the of W64F mutant.

Using 310 nm and 350 nm excitation, tryptophans are not excited the W64F mutant, so the fluorescence from the flavin is the fluorescence intensity of the acceptor in the absence of the donor. Calculating thus the fluorescence intensity of FAD in the dark adapted state for the 7aza-W104W64F mutant is

$$I_A(\lambda_A^{em}) = I_{\lambda exc=310nm}(\lambda_{FAD}^{505nm}) = \frac{I_{\lambda exc=350nm}}{4.71} = \frac{246978}{4.71} = 52437\ cps$$

Therefore the FRET efficiency for the 7aza-W104/W64F mutant, E_{dark} is

$$E_{dark} = \frac{\varepsilon_A(\lambda_D^{ex})}{\varepsilon_D(\lambda_D^{ex})}\left[\frac{I_{AD}(\lambda_A^{em})}{I_A(\lambda_A^{em})} - 1\right] = \frac{860.32}{1{,}013}\left[\frac{78940}{52437} - 1\right] = 0.42$$

Due to the distance dependence of FRET (equation 1) we can estimate a distance

$R = R_0\sqrt[6]{\frac{1-E}{E}} = 16.8\sqrt[6]{\frac{1-0.42}{0.42}}$ =17.7 Å between Trp and FAD in the dark state.

For the 7aza-W104/W64F, Ro=16.8 Å

Calculation of distance R between Trp104 and flavin in the light state of 7aza-W104/W64F mutant.

Using the same methodology, we calculated a distance R between Trp104 and flavin in the light state, R= 9.7Å. In detail, the FRET efficiency for the 7aza-W104/W64F mutant in the light state, E_{light} is calculated as

$$E_{light} = \frac{\varepsilon_A(\lambda_D^{ex})}{\varepsilon_D(\lambda_D^{ex})}\left[\frac{I_{AD}(\lambda_A^{em})}{I_A(\lambda_A^{em})} - 1\right] = \frac{860.32}{1{,}013}\left[\frac{66940}{39377} - 1\right] = 0.59$$

Therefore, the distance between Trp104 and FAD in the light state is calculated to be:

$R = R_0\sqrt[6]{\frac{1-E}{E}} = 8.9\sqrt[6]{\frac{1-0.59}{0.59}}$ =8.3 Å.

Here we used κ^2=0.02. In this case R_0 = 8.9 Å which results in a donor-acceptor distance equal to 8.3 Å.

III. Distance determination from electron transfer theory using transient fluorescence spectroscopy measurements

Using the simple empirical expression known as the Dutton ruler (equation 4)[6]

$$\log k_{ET} = 15 - 0.6R - 3.1\frac{(\Delta G+\lambda)^2}{\lambda} \quad (4)$$

that relates the electron transfer rate (k_{ET}) with the edge-to-edge distance (R in Å) between two reaction centres, assuming a barrierless electron transfer ($\Delta G=-\lambda$), where ΔG is the driving force for the electron transfer between the electron donor and the excited flavin and λ is the reorganization energy, we estimated the distances between the flavin and the tryptophan for the dark-adapted state and the light-adapted state.

Dark W64F: $k_{ET} = \frac{1}{504ps} = 1.98*10^9 s^{-1}$ *and* $R_{dark}=9.5$ Å

Light W64F: $k_{ET} = \frac{1}{11ps} = 90*10^9 s^{-1}$ *and* $R_{dark}=6.7$ Å

Y21FY56FW64F: $k_{ET1} = \frac{1}{200ps} = 5*10^9 s^{-1}$ *and* $R=8.85$ Å

$k_{ET2} = \frac{1}{36.5ps} = 27.4*10^9 s^{-1}$ *and* $R=7.6$ Å

Supplementary Table 1. List of primers for generation of different AppA BLUF constructs.

W64F forward	GGGCGTCTTCTTCCAGTTCCTCGAAGGCCGCCCCG
W64F reverse	CGGGGCGGCCTTCGAGGAACTGGAAGAAGACGCCC
W104A forward	CGCCGCTTTGCGGGAGCGCACATGCAGCTCTCC
W104 reverse	GGAGAGCTGCATGTGCGCTCCCGCAAAGCGGCG
Y21F forward	TCTGGTTTCCTGCTGCTTCCGCAGCCTGGCGGCCC
Y21F reverse	GGGCCGCCAGGCTGCGGAAGCAGCAGGAAACCAGA
Y56F forward	GACCGGCGCGCTCTTCTTCAGCCAGGGCGTCTTCT
Y56F reverse	AGAAGACGCCCTGGCTGAAGAAGAGCGCGCCGGTC

Figure legends

Figure S1. **A)** Absorption spectra of L-Tyrosine (red line), L-Trp (black line) and 7-aza Trp (blue line) in 50mM Tris, 10 mM NaCl, pH 8.00 and L-Tyrosine (green line) in glycine/NaOH, pH 11.0. **B)** Emission spectra of L-Tyrosine (pH=8.0, λ_{exc}=280 nm) (red line), L-Tyrosine (pH=11.0, λ_{exc}=295 nm) (green line), L-Tryptophan (pH=8.0, λ_{exc}=295 nm) (black line), and 7-aza Tryptophan (pH=8.0, λ_{exc}=310 nm). The emission spectra were scaled for visualization purposes. Note that the quantum yield (φ) is 0.13 for L-Trp, 0.01 for 7-aza Trp and 0.14 for L-Tyr

Figure S2. A) Absorption spectra of W64F AppA BLUF in the dark-adapted state (black) and light-adapted state (red). B) shows the recovery kinetics to the dark-adapted state monitored at 490 nm after illumination at 385 nm.

Figure S3. (A, B) Transient ps fluorescence spectra for the AppA W64F (dark- and light-adapted state) at 0.4 ps, 60 ps, 298 ps, 999 ps and 0.4 ps, 4.9 ps, 9.9 ps, 29.9 ps and 198 ps, respectively and (C) AppA Y21F/Y56F/W64F at 0.4 ps, 27.9 ps, 79.9 ps and 398 ps.

Figure S4. Absorption spectrum of FAD in PBS buffer.

Figure S5. Fluorescence anisotropy decay of the Y21F/Y56F/W64F AppA BLUF mutant

Figure S6. Relative orientations of the transition dipole moments of FAD and Trp104 (pdb:1YRX)

Figure S7. Absorption spectra of the Y21FY56FW64F and the Y21FY56FW64FW104F mutant.

Fig. S1

A

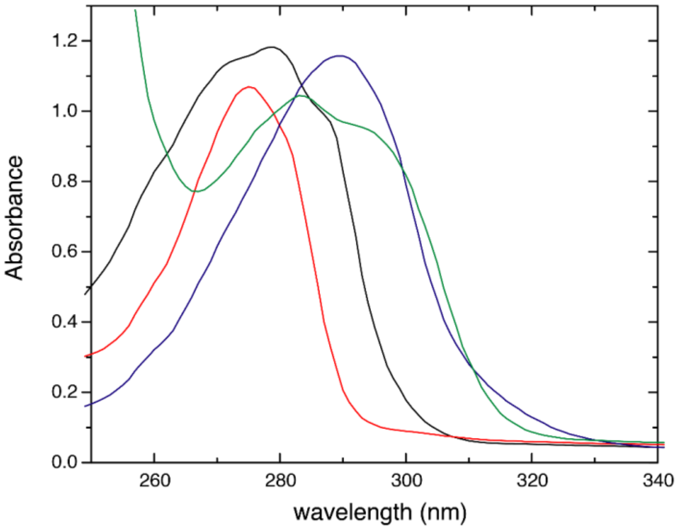
1.2
1.0
0.8
0.6
0.4
0.2
0.0
Absorbance
260
280
300
320
340
wavelength (nm)

B

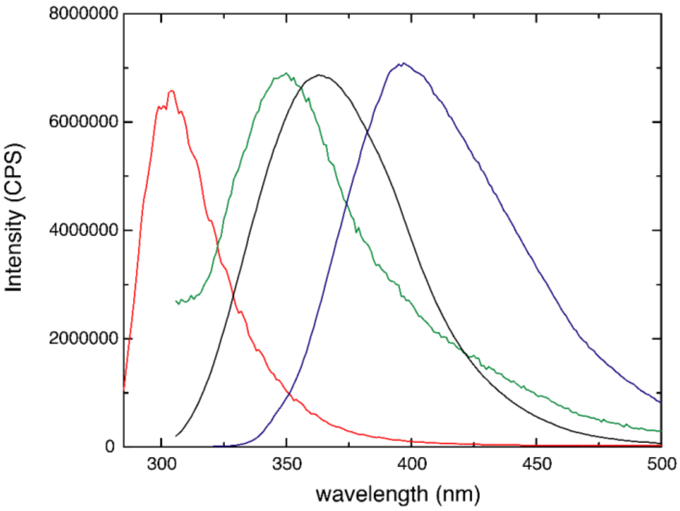
8000000
6000000
4000000
2000000
0
Intensity (CPS)
300
350
400
450
500
wavelength (nm)

Fig. S2

A

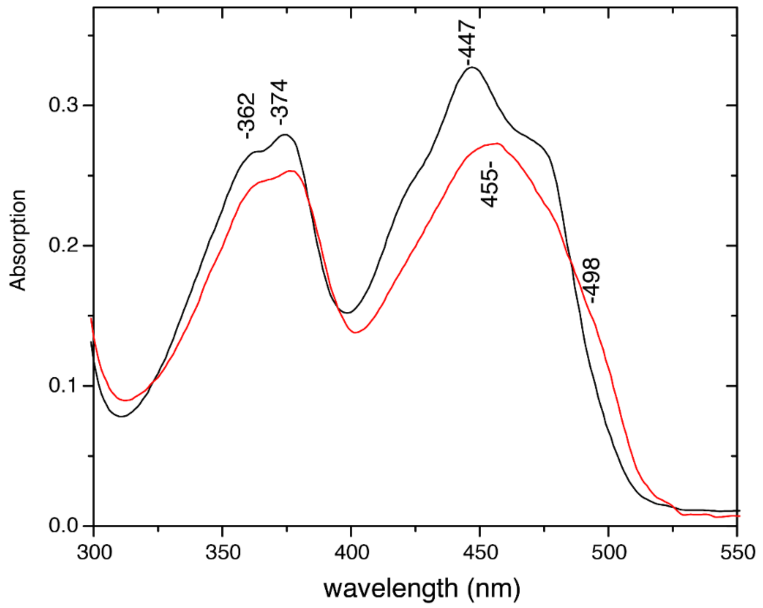
Absorption
0.0
0.1
0.2
0.3
-362
-374
-447
455-
-498
300
350
400
450
500
550
wavelength (nm)

B

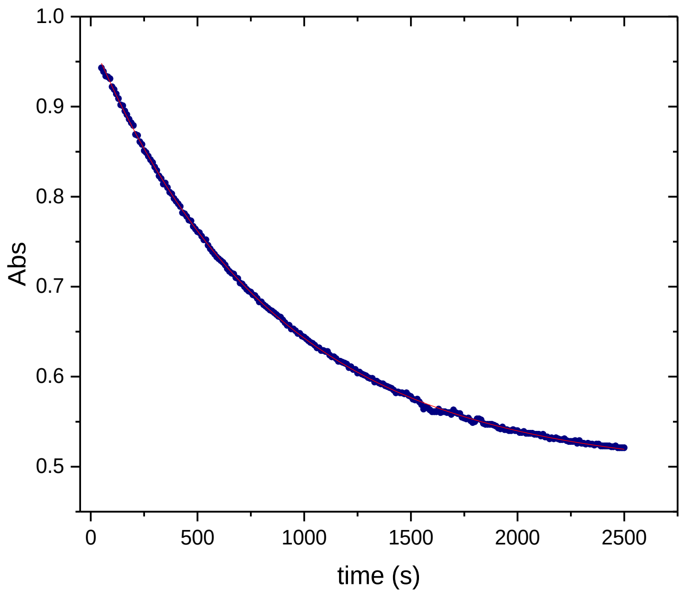
Abs
0.5
0.6
0.7
0.8
0.9
1.0
0
500
1000
1500
2000
2500
time (s)

Fig. S3

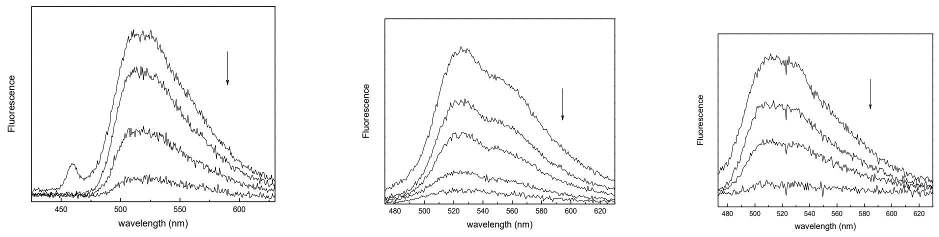

Fig. S4

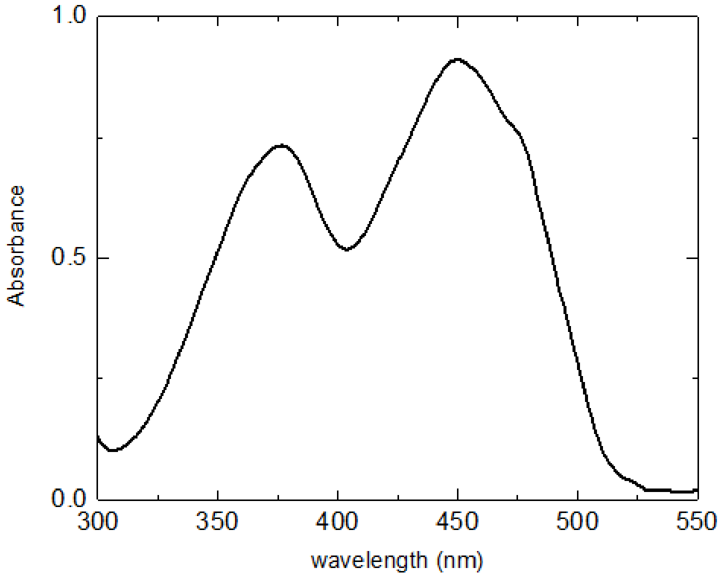

Fig. S5

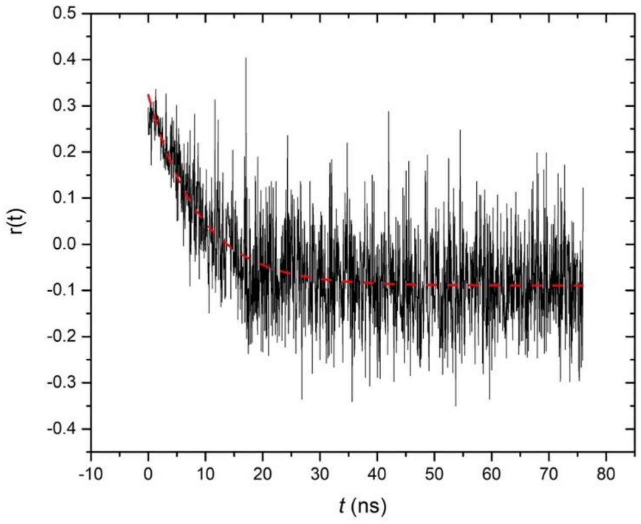

Fig S6.

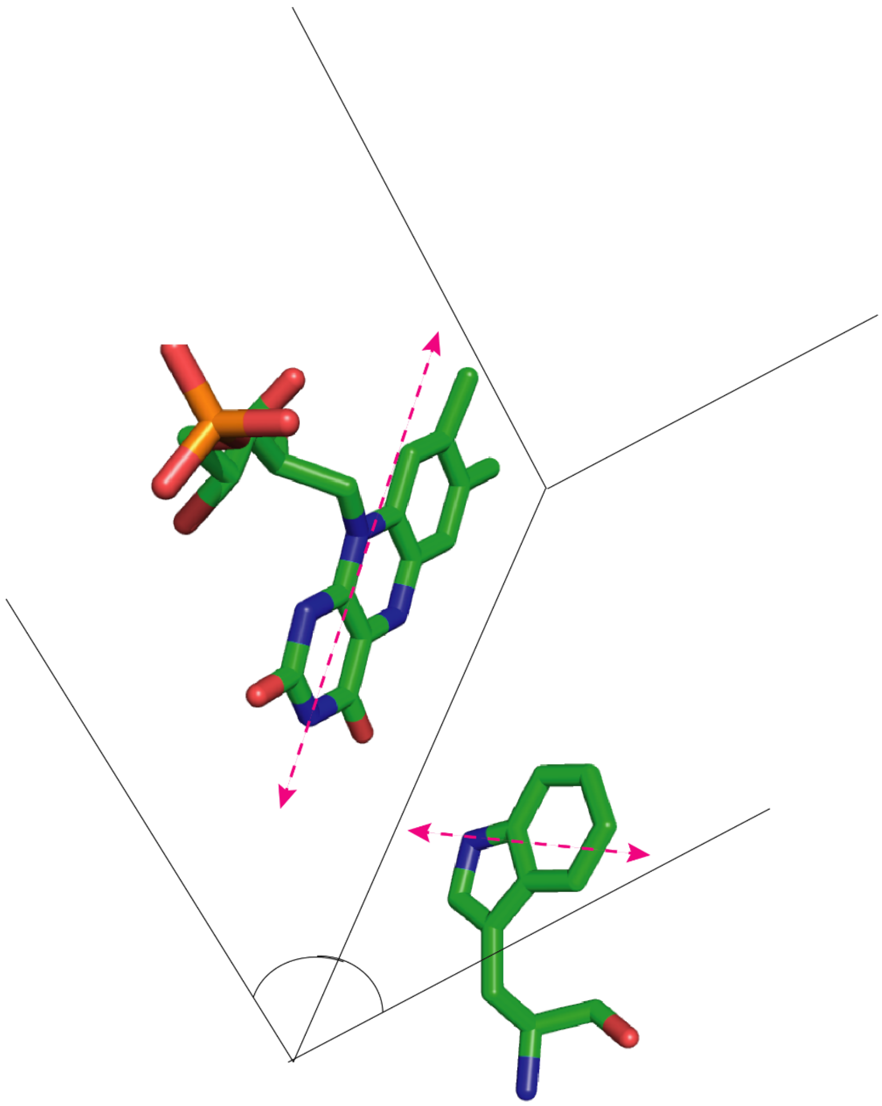

Fig S7.

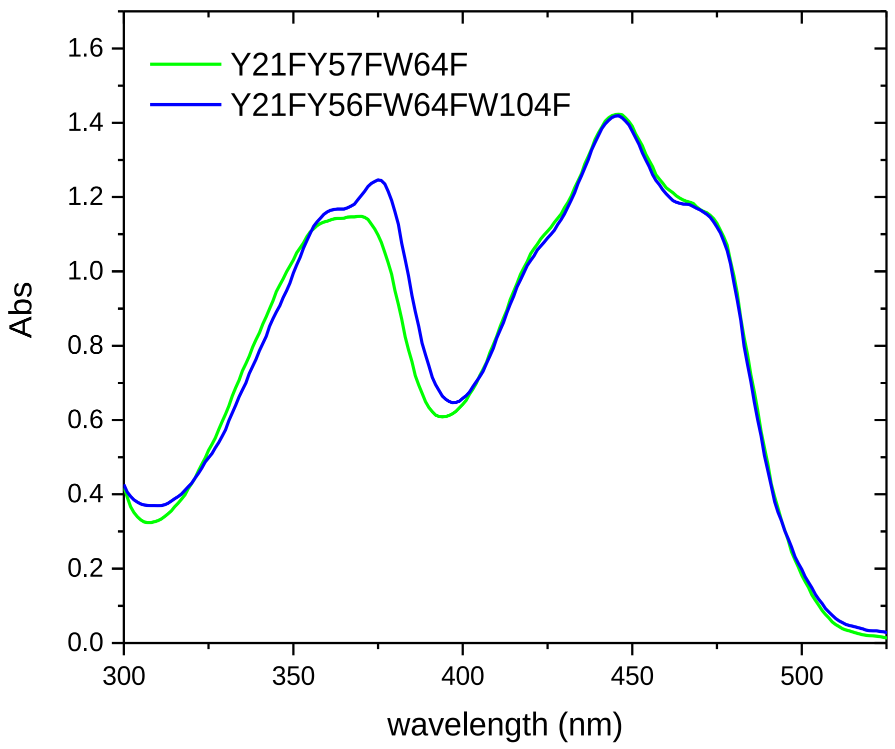

References

1. Joseph R. Lakowicz. *Principles of Fluorescence Spectroscopy*. (Sprimger, 2006).
2. Alexander Ross, J. B., Rusinova, E., Luck, L. A. & Rousslang, K. W. Spectral Enhancement of proteins in vivo incorporation of tryptophan analogues. in *Topics in Fluorescence, Protein fluorescence* **6**, 17–43 (Kluwer Academic Publishers, 2002).
3. Valeur, B. *Molecular Fluorescence: Principles and applications*. (Wiley-VCH Verlag GmbH, 2001).
4. Kraft, B. J. *et al.* Spectroscopic and mutational analysis of the blue-light photoreceptor AppA: A novel photocycle involving flavin stacking with an aromatic amino acid. *Biochemistry* **42**, 6726–6734 (2003).

5. Twine, S. M. & Szabo, A. G. Fluorescent amino acid analogs. *Methods Enzymol* **360**, 104–27 (2003).
6. Dutton, P. L., Page, C. C., Moser, C. C. & Chen, X. Natural engineering principles of electron tunneling in biological oxidation-reduction. *Nature* **402**, 47–52 (1999).
